# Metabonomic Responses of Grazing Yak to Different Concentrate Supplementations in Cold Season

**DOI:** 10.3390/ani10091595

**Published:** 2020-09-08

**Authors:** Jia Zhou, Shuangming Yue, Quanhui Peng, Lizhi Wang, Zhisheng Wang, Bai Xue

**Affiliations:** Animal Nutrition Institute, Sichuan Agricultural University, Chengdu 611130, China; zhoujia1@stu.sicau.edu.cn (J.Z.); yueshuangming@stu.sicau.edu.cn (S.Y.); 14101@sicau.edu.cn (Q.P.); 12825@sicau.edu.cn (L.W.); wangzs@sicau.edu.cn (Z.W.)

**Keywords:** yaks, supplementation, growth performance, serum metabolomics

## Abstract

**Simple Summary:**

The current study employed the nuclear magnetic resonance (NMR) spectroscopy combined with multivariate data analysis to evaluate the effects in supplementation of highland barley (HLB), rapeseed meal (RSM) and HLB plus RSM on serum metabolites in grazing yaks. The findings of the study explored that supplementation with HLB, RSM and HLB plus RSM significantly alleviated the weight loss of growing yak in cold season, and HLB and HLB plus RSM were better than feed RSM. We found that different concentrate supplementations can partially change the metabolic consequences, such as promoted synthesis of lipids and proteins, and supplementation with HBL plus RSM was more effective in promoting lipid and protein deposition and improving energy supply efficiency. The findings of the current experiment may help to explain the body weight changes by supplementation with different concentrates via metabonomic responses.

**Abstract:**

Supplementation plays an important role in reversing the weight loss of grazing yaks during cold season. However, little is known about the effect of supplementation on the serum metabolites of grazing yaks. The objective of this study was to explore the effects of supplementary feeding on average daily gain (ADG) and serum metabolites with nuclear magnetic resonance (NMR)-based metabolomics method in growing yaks during cold season on the Qinghai-Tibetan plateau. Twenty 1.5-year-old female yaks (91.38 ± 10.43 kg LW) were evenly divided into three treatment groups and a control group (CON) (*n* = 5 per group). All the yaks were released to graze during daytime, whereas the yaks in the treatment groups were supplemented with highland barley (HLB), rapeseed meal (RSM), and highland barley plus rapeseed meal (HLB + RSM) at night. The whole experiment lasted for 120 days. Results indicated that the ADG of growing yak heifers was increased by concentrate supplementations, and ADG under HLB and HLB + RSM group was 37.5% higher (*p* < 0.05) than that with RSM supplementation. Supplementary feeding increased the plasma concentrations of total protein (TP), albumin (ALB), and blood urea nitrogen (BUN) of those in the CON group, and concentrations of BUN were higher in the RSM group than in the HLB and HLB + RSM group. Compared with the CON group, serum levels of glutamine, glycine, β-glucose were lower and that of choline was higher in the HLB group; serum levels of lactate were lower and that of choline, glutamate were higher in the HLB + RSM group. Compared with the HLB + RSM group, serum levels of glycerophosphoryl choline (GPC) and lactate were higher, and those of choline, glutamine, glutamate, leucine, *N*-acetyaspartate, α-glucose, and β-glucose were lower in the HLB group; serum levels of citrate, GPC and lactate were higher, and those of 3-Hydroxybutyrate, betaine, choline, glutamate, glutamine, *N*-acetylglycoprotein, *N*-acetyaspartate, α-glucose, and β-glucose were lower in the RSM group. It could be concluded that concentrate supplementations significantly improved the growth performance of growing yaks and supplementation with HBL or HLB plus RSM was better than RSM during the cold season. Supplementation with HBL or HLB plus RSM affected the serum metabolites of grazing yaks, and both treatments promoted lipid synthesis. Supplementation of yaks with HBL plus RSM could improve energy-supply efficiency, protein and lipid deposition compared with HLB and RSM.

## 1. Introduction

Yak (Bos grunniens) is the main livestock species on the Qinghai-Tibet plateau, and is an important constituent of the alpine meadow ecosystem and pastoral industry on the plateau [1]. There are approximately 14 million yaks on Qinghai-Tibet plateau, providing meat, milk, transportation services, and dung for fuel for Tibetan herders on the plateau and other nomadic pastoralists in adjacent regions [2]. The nutritional status of grazing yaks is affected by the nutritional value of natural pastures, which is low in quantity and quality in Qinghai-Tibet plateau during the long cold season (November to the following May) and therefore cannot meet the nutrient requirements of yak [3]. The weight loss of growing yaks in the cold season exceeded 25.6% of the total weight gain during the warm season, with moisture and crude protein accounting for the major components, followed by fat [4]. This led to the traditional yak production cycle in which the yak satiated in summer, fattened in autumn, became thin in winter and died in spring”, which restricted the economic benefits of the yak breeding industry. Many researchers have revealed that the weight loss of yak grazed in winter could be reversed by the supplementation of oat hay [5], highland barley straw [5], rapeseed meal and ground maize [6]—multi-nutrient blocks supplementation [5,7]. Highland barley (HLB) and rapeseed meal (RSM) are the common concentrate supplementations on the Qinghai-Tibet plateau. HLB is a special type of barley and rich in high-starch energy feed. RSM, as a protein feed, is the by-product of rapeseed oil extraction, containing high protein content and amino acids. However, unfortunately, there is less information available about the mechanism of concentrate supplementations for the improvement of yak performance.

Metabolomics can be used to analyze changes in all metabolites in organisms caused by changes in nutritional status [8,9], and therefore it can directly reveal the chemical processes and metabolic state changes in organisms. It is also useful in exploring the metabolic and physiological status of an organisms from the metabolites [10,11]. Metabolic balances may be disturbed by many factors, such as dietary composition [12] and nutrients [13] have been shown to influence metabolism. For example, Tranchida et al. [14] found that animals fed with high sugar and high fat diets perturbed several energy-related metabolites. Another study suggested that zinc chelation, a methionine-hydroxyl analogue, is involved in regulating the metabolism of several amino acids [15]. Nuclear magnetic resonance (NMR) spectroscopy-based metabolomics is one of the techniques commonly used in metabolomics research [16,17,18]. This method can reveal the small molecule metabolites in serum as comprehensive as possible, so as to find a more sensitive marker than traditional clinical indicators [19,20,21]. In this study, we hypothesized that serum metabolites of grazing yaks varied with different concentrate supplementations in cold season, and such variation may be useful in explaining body weight (BW) changes in yaks. The differences of these metabolites were analyzed by ^1^H-NMR spectroscopic profiling and multivariate statistics. The ultimate goal of this study was to evaluate the metabonomic responses to different supplementary feeding in growing yaks in cold season.

## 2. Materials and Methods

### 2.1. Animals, Management and Experimental Treatments

The animal handling procedures were in accordance with the Chinese Guidelines for Animal Welfare. The protocol was approved by the Animal Care and Ethical Committee of Sichuan Agricultural University (#SCAUAC201408-3). The experiment was conducted at the Institute of Animal Science and Veterinary Science of Yushu Tibetan Autonomous Prefecture (Yushu Tibetan Autonomous Prefecture, Qinghai, China). The average temperature during the study period (November to the following March) was −4.9 °C. Twenty 1.5-year-old female yaks (91.38 ± 10.43 kg LW) were randomly divided to three treatment groups and a control group (*n* = 5 per group). Yaks in the control group were grazed in alpine grassland without any supplementation (CON), and yaks in the treatment groups were grazed in the same grassland but supplemented with highland barley (HLB), rapeseed meal (RSM), and highland barley plus rapeseed meal (HLB + RSM). All yaks were released to graze during the daytime for the 120-day trial, while treatment groups were housed and individually received 3 different supplementary feeding treatments at the amount of 0.8 kg/head/d when returned to the enclosure after grazing. The ration compositions and nutrient level of each supplementary feeding are listed in Table 1. All yaks were free to take water throughout the experiment.

Grass samples grazed by experimental yaks were taken on days 0, 60 and 120 of the trial using grass clippers. A total of 50 g mixed-grass sample was dried in a forced-air oven at 65 °C for 24 h and ground through a 1-mm sieve for analysis. The crude protein (CP), ether extract (EE) were analyzed according to the AOAC (2002) [23], and neutral detergent fiber (NDF), acid detergent fiber (ADF) were analyzed according to Van Soest et al. [24].

The body weight of each yak was recorded with a platform scale on two consecutive days before grazing in the morning at the beginning and end of the trial.

### 2.2. Sample Collection

Blood samples were collected between 0800 and 1000 h before grazing in the morning. The samples were collected from the jugular vein into evacuated tubes containing heparin sodium for anti-coagulation on day 0 and day 120 of the trial. Samples were kept on ice then centrifuged at 3000 rpm for 10 min at 4 °C to obtain plasma, which was aliquoted and stored at −20 °C until plasma biochemical parameters analysis. Other blood samples without anticoagulants were taken from the jugular vein into evacuated tubes on day 120 of the trial. These samples were centrifuged at 3000 rpm for 10 min at 4 °C to obtain serum samples, which were immediately transferred to the laboratory and frozen at −20 °C until NMR analysis.

### 2.3. ^1^H NMR Spectroscopic Measurement

Sample preparation and detailed procedures for the determination of serum metabolomics by NMR were followed the published document [25]. Serum samples were thawed at room temperature and homogenized with a vortex mixer. Then, 200 μL of each serum sample was placed in a 1.5 mL tube and mixed well with 400 μL of saline solution containing 75% D_2_O as a field frequency lock. After centrifugation at 12,000 rpm for 10 min at 4 °C, 500 μL of the supernatants were transferred to 5 mm NMR tubes and stored at 4 °C until NMR analysis. In this experiment, there were 5 serum samples in each group for the NMR experiment analysis. The ^1^H NMR spectra for all specimens were acquired at 298 k on a Bruker AVANCE II 600 (Bruker BioSpin GmbH, Karlsruhe, Germany) with a 600.13 Hz, an acquisition time of 2.6 s, the spectral width of 12,335.53 Hz, 5 s relaxation delay with 128 scans collected into 64 k data points, and Carr–Purcell–Meiboom–Gill (recycle delay-90°-(τ − 180°-τ) *n*-acquisition) pulse sequences. One-dimensional (1D) spectra were recorded with the CPMG to suppress water signals and broad protein resonances.

### 2.4. Analysis of NMR Data

^1^H NMR spectra were manually calibrated by stages and baseline with MesReNova 7.1 software (Mestrelab Research S. L., Santiago de Compostela, Spain). Prior to Fourier transformation, the free induction decays (FID) were multiplied by an exponential function with a 1.0 Hz line-broadening factor. The ^1^HNMR spectra were referenced to the L-actate resonance at 1.33 ppm. Each spectrum within the range of 9.0–0.5 was divided into 0.002 ppm bins excluding the residual water region and adjacent spectral peaks from 5.10 to 4.66 ppm and 4.64 to 4.25 ppm. According to the total integrated intensity of each spectrum to normalize the integrals before pattern recognition analysis [26]. The normalized data were subjected to pattern recognition multivariate analysis with the software SIMCA-P+10.0 (Umetrics, Umeå, Sweden), and principal component analysis (PCA) used a data scale conversion method of mean center scaling [27] to generate an overview of the sample distribution and observe possible outliers. Partial least squares (PLS) and orthogonal projection to latent structure (OPLS) were performed on the normalized data, which employed SIMCA-P+ software to find the correlation between NMR data (*X* variable) and other variables (*Y* variable, grouping information). Unit variance scaling was used to be the data scale conversion method of partial least squares-discriminant analysis (PLS-DA) and orthogonal projection to latent structure with discriminant analysis (OPLS-DA) [28]. The quality of the model was tested by the PLS-DA with the 5-fold cross-validation method, and the validity of the model was judged by the cross-validation of RX^2^ and Q^2^ (representing the model-interpretable variables and the predictability of the model, respectively). After that, the effectiveness of the model was further tested by changing the ranking order of classification variable y several times (*n* = 100) to obtain corresponding random Q^2^ values. The parameter of Q^2^ represents the predictive ability of the model. A model was considered significant when the Q^2^ value was significant (*p* < 0.05) through permutation. In order to facilitate the interpretation of results, the metabolites coefficient-coded loading that indicated altered metabolites among the different groups were back-transformed in Excel 2010 (Microsoft, Redmond, WA, USA) and plotted with color-coded absolute coefficient values (|*r*|) of the variables in Matlab script 7.0 (The Mathworks Inc., Natick, MA, USA) to identify the significant contributing metabolites.

On the basis of discriminant significance (*p* < 0.05), the absolute value of the correlation coefficient of the metabolite |r| > 0.811 (r > 0.811, r < −0.811) was set to indicate the significant difference of serum metabolites between different groups. In addition, the red and dark blue color-code was used to indicate high correlation and no correlation, respectively, in the sample class, and interpret the results.

### 2.5. Statistical Analysis

Data from body weight and plasma biochemical parameters which contributed significantly to the classification were statistically analyzed using one-way ANOVA by SPSS 19.0 software (SPSS Inc. Chicago, IL, USA). The differences among the treatment groups were compared by Duncan’s multiple range test. Data were expressed as means ± standard deviation (SD). *p* < 0.05 was considered statistically significant.

## 3. Results

### 3.1. Chemical Compositions of Herbage in Experimental Period

The content of crude protein and crude fat in herbage decreased gradually with the extension of the cold season, and decreased by 35.56% (*p* < 0.05) and 61.77% (*p* < 0.05) at d 120 compared to d 0. However, the content of NDF and ADF increased gradually with the prolonged cold season, and increased by 6.61% (*p* < 0.05), 13.27% (*p* < 0.05) and 7.14% (*p* < 0.05) at d 120 compared to d 0 (Table 2).

### 3.2. Body Weight

Average daily gain in the HLB, RSM and HLB + RSM groups was 61.5% (*p* < 0.05), 38.5% (*p* < 0.05) and 61.5% (*p* < 0.05) higher than that in CON group, respectively, and that in the HLB and HLB + RSM groups increased by 37.5% (*p* < 0.05) compared with the RSM group (Table 3).

### 3.3. Plasma Biochemical Parameters

Concentrations of biochemical parameters in plasma on days 0, 120 were presented in Table 4. Plasma concentrations of TP, ALB and BUN were higher in the HLB, RSM and HLB + RSM groups than that in CON group on d 120 (*p* < 0.05). The BUN concentrations on d 120 in the RSM group were higher compared with the HLB group (*p* < 0.05).

### 3.4. ^1^H NMR Spectra of Serum Samples

**Figure 1:**
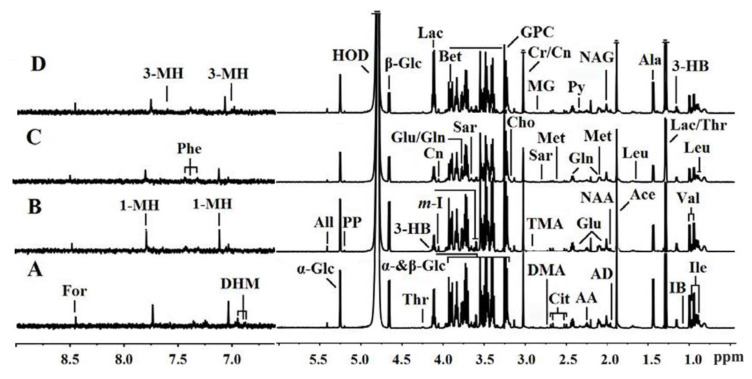
Typical examples of 600-MHz ^1^H NMR spectra (δ0.5–6.0 and δ6.5–9.0) of serum metabolites were obtained from the (**A**) CON group, (**B**) HLB group, (**C**) RSM group, and (**D**) HLB + RSM group respectively. Keys: For: Formate; 1-MH: 1-Methylhistidine; 3-MH: 3-Methylhistidine; Phe: Phenylalanine; 3-HB: 3-Hydroxybutyrate; DHM: 3, 4-Dihydroxymandelate; All: Allantoin; Glc: Glucose; PP: Phophoenolpyruvate; Thr: Threonine; m-I: myo-Inositol; ac: Lactate; Cn: Creatinine; Gln: Glutamine; Glu: Glutamate; Bet: Betaine; Sar: Sarcosine; GPC: Glycerolphosphocholine; Cho: Choline; Cr: Creatine; TMA: Trimethylamine; MG: Methylguanidine; DMA: Dimethylamine; Cit: Citrate; Py: Pyruvate; Met: Methionine; AA: Acetoacetate; NAG: *N*-acetylglycoprotein; NAA: *N*-Acetylaspartate; AD: Acetamide; Ace: Acetate; Ala: Alanine; Val: Valine; Ile: Isoleucine; IB: Isobutyrate.

Typical examples of 600-MHz ^1^H NMR spectra gave a metabolic profile overview in plasma (Figure 1) of growing yaks with different supplementary feeding. Part of the assignments is listed in the figure captions. By visual inspection of the ^1^HNMR spectra, different metabolite patterns were observed among different groups. From Figure 1, it appears that there were significant differences in some chemical shift peaks among the four groups, indicating the differences in metabolite compositions. The resonances from multifarious related metabolites of proteins, lipids and sugars including amino acids, organic acids, glucose and choline are shown in the spectrum of serum samples. In order to obtain more intensive access to metabolic differences analysis in the four groups, we further analyzed the NMR data to reveal the significant differences of identified metabolites using PCA, PLS-DA, and OPLS-DA.

### 3.5. Multivariate Data Analysis of NMR Data

**Figure 2:**
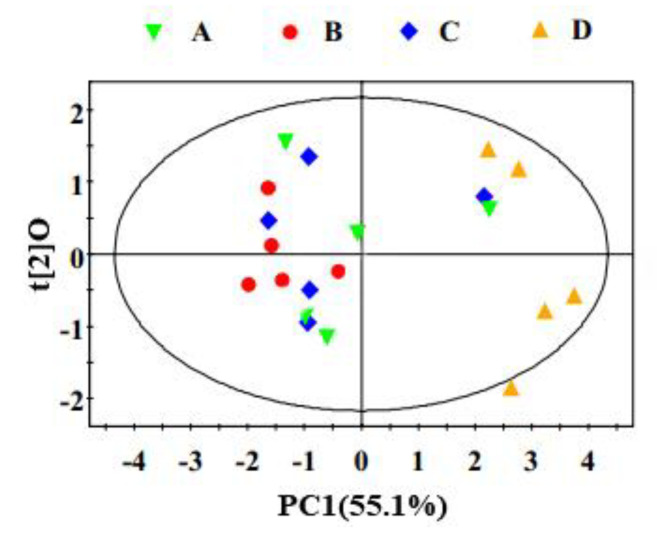
Principle component analysis (PCA) scores plot based on ^1^H NMR spectra of serum was obtained from different groups.

**Figure 3:**
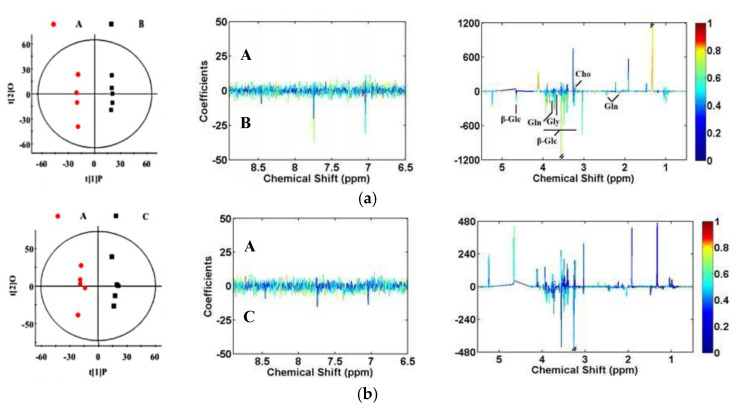

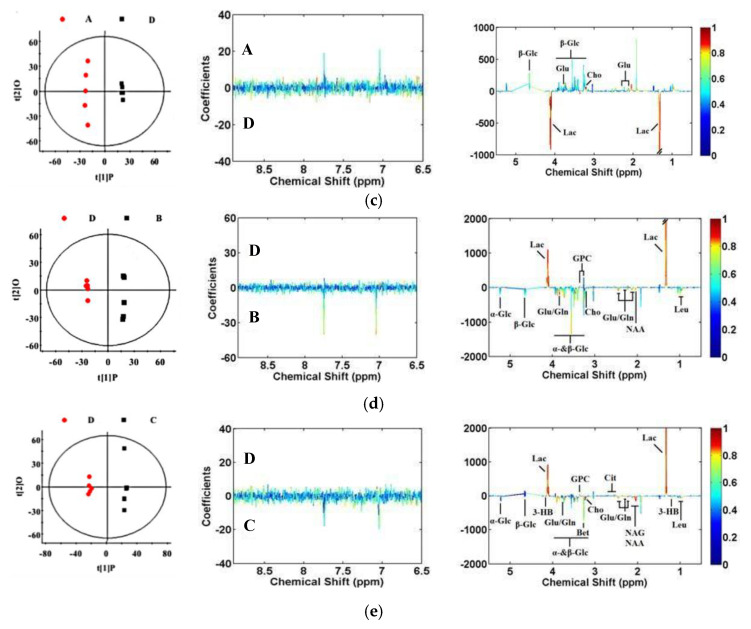
Orthogonal projection to latent structure with discriminant analysis (OPLS-DA) scores plots (left panel) derived from ^1^H NMR spectra of serum and corresponding coefficient loading plots (right panel) were obtained from the (A) CON group, (B) HLB group, (C) RSM group and (D) HLB + RSM group. Score plot of serum: (**a**): CON group vs. HLB group, R^2^X = 22.5%, and Q^2^ = −0.077; (**b**): CON group vs. RSM group, R^2^X = 25.8%, and Q^2^ = −0.480; (**c**): CON group vs. HLB + RSM group, R^2^X = 26.2%, and Q^2^ = −0.241; (**d**): HLB + RSM group vs. HLB group, R^2^X = 24.7%, and Q^2^ = −0.503; (**e**): HLB + RSM group vs. RSM group, R^2^X = 28.4%, and Q^2^ = −0.341. Keys were the same as shown in Figure 1. The color map shows the significance of metabolites variations between the two classes. Peaks in the positive direction indicate that the metabolites are more abundant in the groups in the positive direction of the first principal component. Therefore, peaks in the negative direction indicate that metabolites that are more abundant in the groups in the negative direction of the first principal component.

The PCA of ^1^H NMR data from the serum of yaks in CON, HLB, RSM and HLB + RSM groups were performed, and the scores plot (Figure 2) clearly highlighted four clusters corresponding to the four different groups. The validation of model showed by cross-validation results (Table 5) with PLS-DA of serum spectra. To further understand the significant changes in serum metabolism caused by concentrate supplementation, we compared the metabolic profiles of yaks in four treatments with OPLS-DA. The scores diagram showed significant differences between yaks in CON and HLB group, CON and RSM group, CON and HLB + RSM group, HLB + RSM and HLB group, and HLB + RSM and RSM group (Figure 3). We found that serum concentrations of glutamine, glycine, β-glucose were higher in the CON group compared to the HLB group (*p* < 0.05), whereas serum levels of choline were lower (*p* < 0.05, Figure 3 and Table 6). Serum concentrations of glutamate and choline were higher in the HLB + RSM group compared to the CON group (*p* < 0.05), whereas serum levels of lactate were lower (*p* < 0.05, Figure 3 and Table 6). We also found that serum concentrations of leucine, choline, glycine, glutamine, *N*-acetylglycoprotein (NAG) and glucose were higher in the HLB + RSM group compared to the HLB group (*p < 0.05*), whereas serum levels of GPC and lactate were lower (*p* < 0.05, Figure 3 and Table 6). Serum levels of 3-Hydroxybutyrate (3-HB), betaine, choline, glutamate, glutamine, leucine, NAG, *N*-acetyaspartate and glucose were higher in the HLB + RSM group compared to the RSM group (*p* < 0.05), whereas serum levels of citrate, GPC and lactate were lower (*p* < 0.05, Figure 3 and Table 6).

## 4. Discussion

### 4.1. Chemical Compositions of Herbage and Growth Performance

The results indicated that the yaks in CON group lost most of the BW (16.97%) during the experiment. Under grazing conditions, BW of growing yaks decreased during the cold season [4], and this may be due to the quantity and quality of forages in natural grassland. With the extension of the cold season, the content of CP and EE in herbage decreased, and that of NDF and ADF increased in our study, which is consistent with the previous research results [29]. Therefore, it can be inferred that the BW loss during the cold season was the result of low nutrient contents in forage which were insufficient to meet the maintenance requirement of growing yaks.

The average daily gain (ADG) was higher in the treatment groups than in the CON group (Table 3), which was in accordance with other studies concerning concentrate supplementation of yaks in winter pasture [5,6,7]. We also noted that yaks in the HLB and HLB + RSM groups exhibited better growth performance than those in the RSM groups. Inadequate dietary energy intake may lead to negative energy balance followed by body fat mobilization [30]. Body fat mobilization is the process of breaking down fat to satisfy energy requirements. HLB is a high-starch energy feed that provides more energy than RSM. Hence, it is more efficient for supplementation with HLB to relieve body fat mobilization and BW loss of growing yaks than RSM. These results suggested that the performance of grazing yak could be improved effectively with concentrate supplementation, and HLB is the supreme supplementary feedstuff in winter supplementation for yak.

### 4.2. Plasma Biochemical Parameters

Plasma biochemical parameters are related to the physiological conditions of animals [31], and are useful as indicators of ingested feeds for ruminants [32]. A higher value of plasma TP and ALB means a better quality of protein contained in the diet and the TP concentration decreases in severe protein malnutrition [33,34]. In this study, the concentrations of TP and ALB decreased on day 120 compared with the beginning of trial, could be related to the appreciable low protein intake from dry forage materials. There was a positive effect of concentrate supplementations on plasma concentrations of TP and ALB. BUN is an indicator of dietary amino acid balance and protein metabolism in animals [35]. Under normal conditions, the elevation of BUN level in blood is associated with the excessive breakdown of protein in the body [36]. Thus, the decline in the concentration of BUN in the CON group was consistent with the significant decline in herbage CP content with the extension of the cold season, which is in agreement with the previous report [37]. High energy intake promotes the synthesis of rumen microbial proteins, while the synthesis of rumen microbial proteins is reduced in the absence of energy intake, that produces a great deal of ammonia, then converted to urea in the liver and flow goes to the blood [38,39]. In this study, supplementation with RSM significantly enhanced plasma BUN levels compared with HLB plus RSM. Therefore, it may be helpful to increase the utilization of protein by supplementing protein and energy at the same time.

### 4.3. Blood Metabolomics

In traditional circumstances, yaks graze on pastures without any supplementation during the cold season, which was the case with yaks in CON group in this study. It was found that serum levels of choline were higher in the HLB and HLB + RSM groups compared with those in the CON group. Choline is the precursor of phospholipid such as phosphatidylcholine and sphingomyelin [40], which may contribute to the integrity of intestinal membrane structure [41] and lipid transport metabolism [42]. Excess choline could be used for the synthesis of phosphorylcholine [43], then enhance VLDL synthesis in the liver [44]. The result that serum levels of choline were higher in HLB and HLB + RSM groups could demonstrate that supplementation with HLB and HLB plus RSM promoted lipid synthesis in growing yaks. As we all know, the AMP-activated proteinkinase (AMPK) is activated when energy intake is limited, which promotes catabolism to provide ATP, such as glycolysis, fatty acid oxidation and proteolysis, while inhibiting ATP-consuming anabolism, such as synthesis of fatty acids, protein and glucose (via gluconeogenesis). The deposition of body fat is a dynamic process of fat synthesis and decomposition, depending on the relative rate. We suspect that the starch in HLB was degraded to volatile fatty acids in the rumen for energy, which reduced the oxidative decomposition of fatty acids, resulting in less weight change in the HLB and HLB + RSM groups than in the CON group. It could be related to the inhibition of AMPK signaling pathway in grazing yaks by supplementation with HLB and HLB plus RSM. After prolonged starvation, proteins in some tissues (mainly muscles) are broken down to provide energy for other organs, leading to elevated levels of some amino acids in the serum [45]. Therefore, the plasma concentrations of TP and ALB were higher in treatment groups than those in CON group, which was not only affected by the protein intake in supplementary feeding, but also may reduce the degradation of body protein. The serum levels of glutamine and glycine were lower in the HLB group than the CON group, possibly due to the reduction in body protein decomposition by the supplementation of HLB. As the central organ for the regulation of energy distribution in ruminants, the liver is responsible for distributing and regulating the energy supply to match the normal operation of all parts of the body. The liver converts non-sugar precursors (including lactic acid, glycerol, and glycosaminoacids) to glucose by gluconeogenesis [46]. Glutamine and glycine are both precursors of the glucose synthesis in the process of gluconeogenesis [47]. In this study, the serum concentrations of glycine, glutamine, and β-glucose were lower by supplementation with HLB compared with CON group. It could be suggested that the gluconeogenesis of natural grazing yaks without supplementation was enhanced to gain more glucose. Deng et al. [48] reported that HLB were rich in ferulic acid, naringin, and catechin, which showed favorable hypoglycemic activity via enhancing glucose consumption and glycogen synthesis, which may cause the decrease in serum glucose levels in HLB group. Lactate is an end product of anaerobic glycolysis in the body [49], which is an inefficient method of energy supplementation compared with that of aerobic oxidation. In this study, the serum concentrations of lactate were lower by supplementation with HLB plus RSM compared with CON group. It could be concluded that the efficiency of glucose metabolism in plateau grazing yaks was low, and supplementation with HLB plus RSM could cause glucose catabolism in the direction of high energy-supply efficiency.

The effects of a single concentrate supplementations and the compounds of two concentrates on serum metabolites in growing yak were further compared. We found the serum concentrations of lactate were lower in HLB + RSM group compared with HLB and RSM groups, which demonstrated that supplementation with HLB plus RSM could improve energy-supply efficiency rather than supplementation with HLB or RSM. Serum levels of choline were higher and GPC were lower in HLB + RSM group compared with HLB group. GPC is synthesized from choline in mammals, and inhibits the activity of lysophospholipase, which participates in cell signal transduction and enhances fat oxidation by catalyzing the hydrolysis of lysophospholipids [50,51]. Accordingly, a reduced ratio of choline to GPC implies a metabolic change from choline to GPC in the HLB group. The high choline content, together with the low content of GPC in HLB + RSM group could be associated with fat synthesis. The serum concentrations of glucose and glucogenic amino acids (glutamate and glutamine) were lower by Supplementation with HLB compared with HLB plus RSM, which demonstrated that gluconeogenesis was inhibited in the HLB group. These changes may be affected by a higher intake of HLB in HLB group. Glutamine is a building block of peptides and proteins [52], and glutamine promotes protein synthesis in mammals [53] and inhibits protein breakdown [54]. Leucine is mainly used as a protein synthesizer [55] and widely applied for the estimation of whole-body protein synthesis in heifers [56]. Serum levels of glutamine and leucine were higher in the HLB + RSM group, which may indicate that supplementation with HLB plus RSM had significant effects on protein synthesis compared with HLB. Data of serum metabolites suggested supplementation with HLB plus RSM could improve energy-supply efficiency and synthesis of fat and protein in growing yaks compared with HLB.

The alterations of some metabolites in the RSM group were similar to those in HLB group compared with HLB + RSM group, such as lactate, glutamate, glutamine, glucose, choline and GPC; therefore, HLB plus RSM supplementation favored energy-supply efficiency and gluconeogenesis in grazing and growing yaks. Meanwhile, dietary supplementation with HLB plus RSM could increase the levels of 3-HB, betaine and NAG compared with RSM. 3-HB is the main ketone body generated from lipolysis in liver mitochondria [57] and synthesized in ruminant liver from either non-esterified fatty acid or butyrate arising from rumen fermentation. The decrease in glucose in serum could promote the uptake of 3-HB in tissues, which reduce the utilization rate of energy substrate and decrease the concentration of serum 3-HB [46]. The high choline and 3-HB contents together with the low GPC content in HLB + RSM group could be associated with the fat deposition and synthesis. Betaine is generated by oxidation of choline, plays an important role in lipid metabolism [58] and protein synthesis [59]. Serum concentrations of betaine were higher in HLB + RSM group indicated that supplementation with HLB plus RSM is beneficial for synthesis of fat and protein in growing yaks compared with RSM. These are consistent with the BW change of the yaks in HLB + RSM group.

In conclusion, altered metabolism induced by concentrate supplementation was identified, including alterations in glucose, lipid, and protein synthesis. During the cold season, the weight loss of grazing yaks was caused by insufficient nutrient intake to mobilize body fat and body protein, while supplementation with HLB could promote lipid synthesis and decrease proteolysis, and supplementation with HLB plus RSM could improve energy supply efficiency and promote lipid synthesis. Compared with HLB and RSM groups, supplementation with HLB plus RSM could improve energy supply efficiency, promote lipid and protein synthesis, and decrease lipolysis. Chumpawadee et al. [60] and Krehbiel et al. [61] suggested that synchronous release of dietary energy and nitrogen has a positive effect on growth performance, microbial protein synthesis and nitrogen utilization in ruminants, which agreed well with ADG in HLB plus RSM-supplemented yaks, which displayed higher ADG than the RSM-supplemented yaks.

## 5. Conclusions

The serum metabolic profiles were clearly isolated according to diet type, and these results revealed the usefulness of the routine use of metabolomics in nutritional and supplementation studies, which is useful for distinguishing individuals based on concentration intake. Metabolomics studies have been demonstrated to be useful in describing metabolic characteristics associated with the dietary supplementation of energy or protein feeds.

Different concentrate supplementations are helpful for alleviating the weight loss of grazing yaks in cold season and changing some of the metabolic consequences—such as glucose, lipid, and protein synthesis. Our results support that supplementation with HBL plus RSM was more effective in promoting lipid and protein deposition and improving energy supply efficiency.

## Figures and Tables

**Table 1:** Ingredients and chemical composition of supplementary concentrations (air-dry basis).

Items	HLB	RSM	HLB + RSM
Ingredient composition (%)			
Highland barley	96.60	0	87.00
Rapeseed meal	0	99.0	9.60
NaCl	1.00	1.00	1.00
Dicalcium phosphate	2.40	0	2.40
In total	100.00	100.00	100.00
Chemical composition (%)			
NEmf ^1^ (MJ/kg)	6.93	6.70	6.90
Dry matter	90.33	91.25	90.74
Crude protein	10.41	36.03	12.90
Neutral detergent fiber	54.74	34.15	51.95
Acid detergent fiber	8.26	26.58	10.12
Calcium	0.68	0.72	0.73
Phosphorus	0.67	1.04	7.70

^1^ NEmf was referenced from NY/T815-2004 [22], the others were measured values.

**Table 2:** Compositions of herbage in different experiment times.

Items	Days		
	0	60	120
Nutrition levels (%, DM)			
Crude protein	7.79 ± 0.45 ^c^	5.60 ± 0.17 ^b^	5.02 ± 0.58 ^a^
Ether extract	3.61 ± 0.31 ^c^	2.24 ± 0.28 ^b^	1.38 ± 0.14 ^a^
Neutral detergent fiber	50.56 ± 1.55 ^a^	51.89 ± 1.50 ^ab^	53.90 ± 0.95 ^b^
Acid detergent fiber	32.26 ± 1.52 ^a^	33.95 ± 1.14 ^ab^	36.54 ± 1.26 ^b^

Data with different small letter superscripts within the same row are significantly different (*p* < 0.05).

**Table 3:** Effects of different concentrate supplementations on body weight of growing yaks.

Item	CON	HLB	RSM	HLB + RSM
Initial weight (kg)	93.7 ± 9.2	93.6 ± 12.3	92.5 ± 10.1	85.7 ± 10.1
Final weight (kg)	77.8 ± 8.5	87.4 ± 11.7	83.2 ± 11.0	79.5 ± 9.9
Body weight change (kg)	−15.9 ± 3.6 ^a^	−6.2 ± 1.1 ^b^	−9.3 ± 1.8 ^b^	−6.2 ± 1.8 ^b^
Average daily gain (g/d)	−133 ± 32 ^a^	−51.7 ± 9.8 ^c^	−77.5 ± 17.3 ^b^	−51.6 ± 17.2 ^c^
Average concentrate intake (g/d)	—	800	800	800

Data with different small letter superscripts within the same row are significantly different (*p* < 0.05).

**Table 4:** Effects of supplementary different feeds on blood biochemical parameters of growing yaks.

Item	Days	CON	HLB	RSM	HLB + RSM
TP (g/L)	0	65.4 ± 2.7	68.2 ± 5.9	66.2 ± 5.2	65.8 ± 1.8
	120	53.5 ± 7.1 ^a^	59.9 ± 7.1 ^b^	64.5 ± 4.6 ^b^	62.6 ± 2.7 ^b^
ALB (g/L)	0	41.5 ± 3.5	43.6 ± 4.1	40.9 ± 2.3	43.2 ± 2.6
	120	35.2 ± 1.9 ^a^	39.6 ± 2.5 ^b^	39.2 ± 2.1 ^b^	41.3 ± 2.4 ^ab^
BUN (mmol/L)	0	4.7 ± 0.3	4.7 ± 0.5	4.5 ± 0.9	4.6 ± 0.5
	120	2.3 ± 0.6 ^a^	4.7 ± 1.0 ^b^	5.9 ± 1.0 ^c^	4.8 ± 0.6 ^bc^

Data with different small letter superscripts within the same row are significantly different (*p* < 0.05).

**Table 5:** Cross-validation results of partial least squares-discriminant analysis (PLS-DA) model summary for discriminating in different groups.

Groups	Intercept	Groups	Intercept
A vs. B	R^2^ = (0, 0.997); Q^2^ = (0, 0.519)	D vs. B	R^2^ = (0, 0.995); Q^2^ = (0, 0.219)
A vs. C	R^2^ = (0, 0.996); Q^2^ = (0, 0.345)	D vs. C	R^2^ = (0, 0.990); Q^2^ = (0, 0.230)
A vs. D	R^2^ = (0, 0.995); Q^2^ = (0, 0.372)		

Cross-validation results of PLS-DA model summary for discriminating for groups CON (A), HLB (B), RSM (C), HLB + RSM (D).

**Table 6:** OPLS-DA coefficients derived from the NMR data of metabolites in serum obtained from different groups of growing yaks.

Metabolites ^1^	Identification (ppm) and Multiplicity ^2^	Correlation Coefficients (r) ^3,4^				
		A vs. B	A vs. C	A vs. D	D vs. B	D vs. C
ffff3-HB	1.20 (d),2.31 (dd), 4.16 (m)	-	-	-	-	0.844
Betaine	3.27 (s), 3.90 (s)	-	-	-	-	0.897
Choline	3.20 (s)	−0.820	-	−0.903	0.873	0.832
Citrate	2.53 (d), 2.70 (d)	-	-	-	-	−0.818
Glutamate	2.12 (m), 2.35 (m), 3.78 (t)	-	-	−0.853	0.840	0.902
Glutamine	2.14 (m), 2.45 (m), 3.78 (t)	0.876	-	-	0.845	0.902
Glycine	3.56 (s)	0.861	-	-	-	-
GPC ^4^	3.23 (s), 3.36 (s)	-	-	-	−0.830	−0.846
Lactate	1.33 (d), 4.11 (q)	-	-	0.903	−0.968	−0.919
Leucine	0.94 (d), 0.97 (d), 1.66 (m)	-	-	-	0.854	-
NAG	2.04 (s)	-	-	-	-	0.873
NAC	2.02 (s), 2.53 (m), 2.70 (m)	-	-	-	0.826	0.876
α-Glucose	3.42 (t), 3.54 (dd), 3.71 (t), 3.73 (m), 3.84 (m), 5.24 (d)	-	-	-	0.877	0.917
β-Glucose	3.25 (dd), 3.41 (t), 3.46 (m), 3.49 (t), 3.90 (dd),	0.837	-	-	0.940	0.823

^1^ 3-HB: 3-Hydroxybutyrate; GPC: glycerophosphoryl choline; NAG: *N*-acetylglycoprotein; NAA: *N*-Acetylaspartate. ^2^ Multiplicity: s, singlet; d, doublet; t, triplet; q, quartet; dd, doublet of doublets; m, multi plet; br, broad resonance. ^3^ Correlation coefficients for groups CON (A), HLB (B), RSM (C), HLB + RSM (D). ^4^ Correlation coefficients, positive and negative correlation in the concentrations are showed by positive and negative signs. │*r*│ > 0.811 was regard as statistical significance based on the discrimination significance at the level of *p* = 0.05 and df (degree of freedom) = 5. “-” means the correlation coefficient │*r*│ < 0.811.
